# Focal Alveolar Hemorrhage Secondary to Hydralazine-Associated Antineutrophilic Cytoplasmic Antibody Vasculitis

**DOI:** 10.7759/cureus.42031

**Published:** 2023-07-17

**Authors:** Jose M Martinez Manzano, Simone A Jarrett, Kevin Bryan Lo, Mitali Sen, Irene J Tan

**Affiliations:** 1 Internal Medicine, Einstein Medical Center Philadelphia, Philadelphia, USA; 2 Rheumatology, Einstein Medical Center Philadelphia, Philadelphia, USA

**Keywords:** hydralazine-associated vasculitis, hemoptysis, focal alveolar hemorrhage, pulmonary capillaritis, anca vasculitis

## Abstract

Hydralazine is rarely associated with antineutrophilic cytoplasmic antibody (ANCA) vasculitis. In the appropriate clinical scenario, such as in a patient with pulmonary, renal, or cutaneous manifestations, finding antibodies against nuclear and cytoplasmic neutrophil antigens may suggest drug-induced vasculitis after exposure to hydralazine. We present the case of an elderly man diagnosed with focal alveolar hemorrhage with elevated concentrations of anti-myeloperoxidase antibody, anti-proteinase-3 antibody, and antinuclear antibodies in the setting of prolonged hydralazine therapy. We observed a rapid clinical improvement with hydralazine discontinuation and systemic corticosteroids. We did not observe further disease activity while on mycophenolate mofetil six months later.

## Introduction

Hydralazine is a direct-acting vasodilator used for the treatment of hypertension for more than 70 years [1]. Hydralazine has been associated with drug-induced lupus erythematosus in up to 5%-10% of patients and more rarely with antineutrophilic cytoplasmic antibody (ANCA)-associated vasculitis (AAV) [2]. The first case of hydralazine-AAV was reported in the 1980s [3]. The clinical features of hydralazine-AAV resemble those of idiopathic AAV [4]. The lungs seem affected in 19% to 92% of cases, manifesting as hemoptysis, pulmonary infiltrates, and pleural effusions [5]. In contrast to idiopathic AAV, patients with hydralazine-AAV often have autoantibodies against multiple (nuclear and cytoplasmic) neutrophil antigens [4]. Due to limited evidence, there is no standardized therapy for hydralazine-AAV, and most patients tend to be treated with aggressive immunosuppression to induce remission, similar to idiopathic AAV [4,6]. We aim to describe, in accordance with the CARE guidelines (for CAse REports) [7], an uncommon presentation of hydralazine-AAV as focal pulmonary hemorrhage in an elderly man and to review the literature.

## Case presentation

A 69-year-old Hispanic man presented to the emergency department with progressive dyspnea and one day of hemoptysis. He denied recent fevers, chills, sputum production, chest pain, weight loss, night sweats, epistaxis, hematemesis, melena, or hematuria. His medical history was significant for heart failure with improved ejection fraction secondary to ischemic cardiomyopathy, atrial fibrillation, chronic kidney disease stage 3, and hypertension. His family history was noncontributory, and his social history was negative for smoking, alcohol use, and illicit drug use. He was on apixaban, aspirin, carvedilol, hydralazine, isosorbide mononitrate, atorvastatin, and repaglinide.

**Figure 1:**
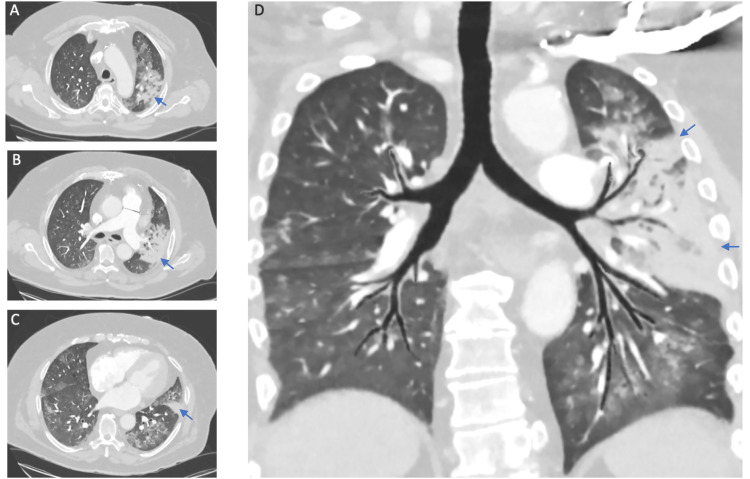
Chest computed tomography angiography Confluent area of consolidation with an air bronchogram in the left upper lobe and diffuse ground glass opacities. A-C: axial views in the upper, medium, and lower lung zones; D: coronal view

On admission, he was afebrile, tachycardic, tachypneic, and normotensive. His oxygen saturation was 91% in ambient air. Lung auscultation revealed bilateral crackles, worse on the left side. A chest computed tomography angiography (CTA) showed a left upper lobe radiopacity and no evidence of pulmonary embolism (Figure 1).

Laboratory analysis revealed low hemoglobin at 9.6 g/dl (reference range, 14 to 18 g/dl), a normal white blood cell count, and platelets. His creatinine was at his baseline level of 2.25 g/dl (reference range, 0.7 to 1.2 g/dl). Coagulation studies were normal. Urinalysis showed microscopic hematuria with 31-50 red blood cells per high-power field (reference range, 0-5) with no proteinuria. His complement C3 concentration was 76 mg/dL (reference range, > 82 mg/dL), C4 was normal, and antinuclear antibody (ANA) screening was positive (titer was 1:320 with a homogeneous pattern). A urine drug screening was negative.

**Figure 2:**
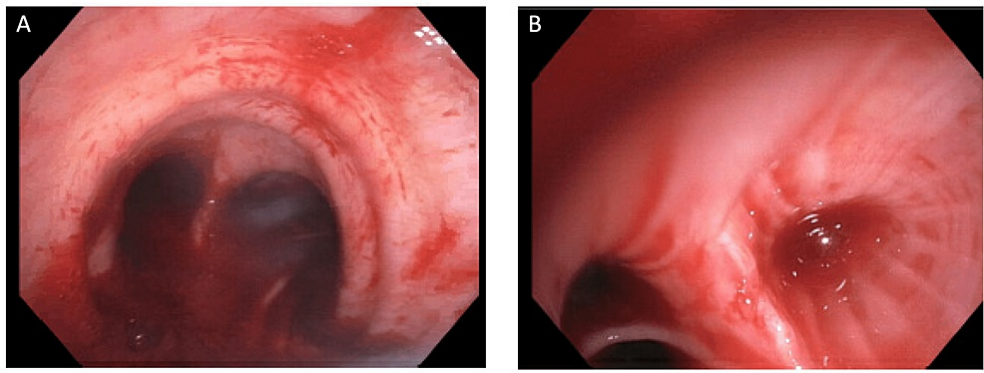
Flexible bronchoscopy Bright red blood was visualized at the level of the carina (A), and active oozing was noted coming from the left mainstem bronchus, specifically the inferior segment of the lingula (B). No source of bleeding was visualized in the upper airway.

Flexible bronchoscopy was performed due to ongoing hemoptysis and a localized source of bleeding on chest CTA, which revealed endobronchial bleeding coming from the left inferior lingula segment (Figure 2), which did not resolve with endobronchial cold saline lavage or with endobronchial wedging.

**Figure 3:**
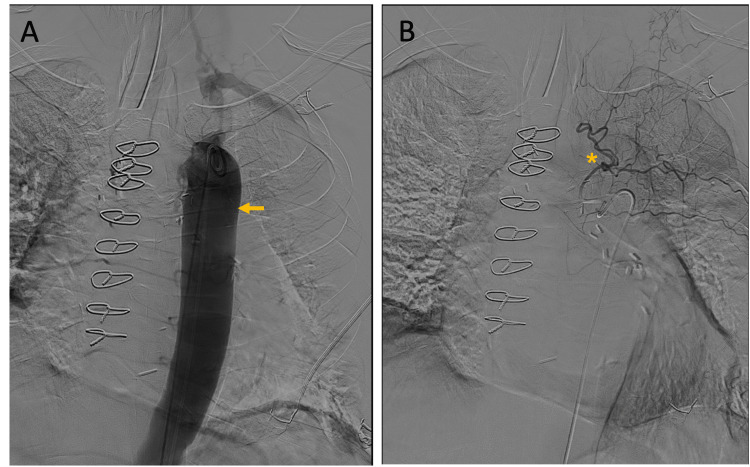
Descending thoracic aortogram A: descending aorta (arrow); B: left upper lobe bronchial vessels (*). Showing no evidence of intravascular contrast extravasation.

A descending thoracic aortogram showed bilaterally normal bronchial and intercostal arteries, with no evidence of large or medium vessel (arterial) bleeding (Figure 3).

The diagnosis of focal alveolar hemorrhage (FAH) was established.

His home anticoagulation and antiplatelet therapies were discontinued, and he was promptly treated with intravenous methylprednisolone 100 mg daily for three days, followed by an oral taper. Further rheumatologic testing was available on day 10, showing an elevated titer of p-ANCA >1:640 (reference range, < 1:20), an elevated concentration of ANCA-myeloperoxidase (MPO) >800 nmol/min/mL (reference range, <1 nmol/min/mL), and an elevated concentration of anti-proteinase 3 (PR3) 27.1 IU/mL (reference range, <1 U/mL). The concentration of anti-double-stranded DNA was mildly elevated at 9 U/ml (reference range, <5 U/ml). The remaining tests were negative. At this point, we had a strong clinical suspicion of hydralazine as the trigger of ANCA vasculitis, and we discontinued hydralazine.

He continued to have intermittent episodes of mild hemoptysis that self-resolved on day 12, and he was discharged home on day 14. His blood pressure was controlled by adding nifedipine to his baseline regimen upon discharge. At the one-month follow-up, repeated blood analysis showed a down-trending concentration of ANCA-myeloperoxidase (MPO) at 404.7 nmol/min/mL (reference range, <1 nmol/min/mL) and ANCA-PR3 at 14.6 IU/mL (reference range, <1 IU/mL). At the two-month follow-up, we transitioned him to mycophenolate mofetil. Repeat chest radiographs at two- and six-month follow-ups showed interval resolution of pulmonary infiltrates.

## Discussion

We present the case of an elderly man with hemoptysis secondary to FAH, likely mediated by pulmonary capillaritis (a small vessel vasculitis) owing to ANCA antibodies. Although ANCA vasculitis most commonly presents as bilateral pulmonary infiltrates, unilateral bleeding, as observed in our patient, is still a possible (but atypical) presentation [8,9]. Furthermore, in the setting of hydralazine therapy, the presence of ANCA multi-antigenicity (positive MPO and PR3) and the particularly elevated ANCA-MPO concentration (800-fold greater than normal) made hydralazine-AAV the most plausible diagnosis [5].

Multiple drugs have been associated with AAV, of which hydralazine is the most common, followed by propylthiouracil, thiamazole, and others [2]. Illicit drugs such as cocaine contaminated with levamisole are also known culprits of AAV, which was ruled out in our patient with a negative urine drug screening [10]. In the present case, the patient was exposed to hydralazine for at least three years before presentation, with a daily dose ranging from 150-300 mg/day, similar to the cumulative doses documented in prior studies [6].

Multiple pathophysiologic mechanisms have been postulated with hydralazine-AAV; one of the plausible mechanisms hypothesizes that the binding of hydralazine to MPO causes neutrophil cytotoxicity with subsequent exposure of sequestered intracellular antigens, resulting in the production of autoantibodies against multiple (nuclear and cytoplasmic) antigens [11]. Another mechanism consists of the excessive expression of neutrophilic granular proteins (MPO and PR-3) mediated by hydralazine-induced DNA demethylation, a mechanism by which hydralazine was studied as an antineoplastic agent [10].

Hydralazine-AAV affects the lungs in 19% to 92% of cases [5]. As pulmonary involvement is an independent predictor of mortality in idiopathic ANCA vasculitis, pulmonary involvement in hydralazine-AAV is of clinical relevance. It may serve as a guide for the therapeutic approach [12-14]. A recent literature review of 35 patients with hydralazine-AAV with pulmonary involvement showed an inpatient mortality rate of 29% despite most patients being aggressively treated with corticosteroids, cyclophosphamide, and rituximab [15].

In a case series of 10 patients with hydralazine-AAV, the most common pulmonary manifestation was hemoptysis, as observed in our case [5]. Another case series of 12 patients with hydralazine-AAV reported bilateral pulmonary infiltrates and pleural effusions as the most common pulmonary manifestations, whereas none reported hemoptysis [6]. To our knowledge, this is the first case of hydralazine-AAV manifesting as FAH that has been reported.

Our patient was treated with high-dose corticosteroids and hydralazine discontinuation. In contrast to prior reports, we did not use induction agents such as cyclophosphamide, rituximab, or plasmapheresis to induce remission [4-6, 16]. The role of maintenance therapy is also unclear in these patients. Still, due to pulmonary involvement (and concerns for poor outcomes), we maintained the patient on prolonged immunosuppression with mycophenolate mofetil at two months of follow-up, after a prednisone taper. This therapeutic approach seemed successful, and no evidence of recurrent pulmonary disease was observed on follow-up. Ultimately, the plan is to stop immunosuppression and monitor clinically.

## Conclusions

In summary, we reported an atypical presentation of hydralazine-AAV manifesting as FAH that was successfully treated with hydralazine discontinuation and high-dose corticosteroid therapy. Further studies are needed to establish a standard approach to treatment among these patients based on disease severity.
